# Assessment of Hepatoprotective Effect of Chokeberry Juice in Rats Treated Chronically with Carbon Tetrachloride

**DOI:** 10.3390/molecules25061268

**Published:** 2020-03-11

**Authors:** Hanna Piotrowska-Kempisty, Michał Nowicki, Jadwiga Jodynis-Liebert, Monika Kurpik, Małgorzata Ewertowska, Teresa Adamska, Jan Oszmiański, Małgorzata Kujawska

**Affiliations:** 1 Department of Toxicology, Poznan University of Medical Sciences, 60-631 Poznań, Poland; hpiotrow@ump.edu.pl (H.P.-K.); liebert@ump.edu.pl (J.J.-L.); m.kurpik@wp.pl (M.K.); mewertow@ump.edu.pl (M.E.); tadamska@ump.edu.pl (T.A.); 2 Department of Histology and Embryology, Poznan University of Medical Sciences, 60-781 Poznan, Poland; mnowicki@ump.edu.pl; 3 Department of Fruit, Vegetable and Cereal Technology, Environmental and Life Science University, 51-630 Wrocław, Poland; jan.oszmianski@upwr.edu.pl

**Keywords:** chokeberry, liver fibrosis, oxidative stress, silymarin

## Abstract

The aim of this study was to compare the protective effects of chokeberry juice and silymarin against chemical-induced liver fibrosis in rats. Liver fibrosis was induced by CCl_4_ administered two days a week for six weeks. Two groups of rats were co-treated with chokeberry juice, 10 mL/kg/day. or silymarin as a positive control, 100 mg/kg/day for six weeks. Hepatic lipid peroxidation was suppressed by 50% and the activity of hepatic antioxidant enzymes was increased by 19%–173% in rats co-treated with CCl_4_ and substances tested as compared to rats administered CCl_4_ alone. Hepatic hydroxyproline was decreased by 24% only in rats treated with silymarin. The messenger RNA (mRNA) expression levels of fibrosis-related molecules, procollagen I, α-SMA, TIMP-1, TGFβ, and TNFα, which were significantly increased in the liver of CCl_4_-treated rats, were not modulated by substances tested. Histological evaluation revealed a slight protective effect of silymarin against fibrosis. However, in CCl_4_ + chokeberry-treated rats, the density of vacuolated hepatocytes was significantly lower than that in silymarin administered animals. Chokeberry juice did not demonstrate an antifibrotic effect in the applied experimental model of fibrosis, and the effect of the known antifibrotic agent, silymarin, was very limited.

## 1. Introduction

Fibrosis progressing to cirrhosis is a major complication of various chronic liver diseases such as alcoholic, viral, and autoimmune hepatitis. Morbidity and mortality due to advanced cirrhosis are comparable to those of malignancies [1]. Hepatic fibrosis results from the disequilibrium between synthesis and degradation of extracellular matrix (ECM) components. Due to various triggers, liver macrophages (Kupffer cells) and other inflammatory cells are activated, which leads to the production of cytokines including the profibrogenic cytokines such as platelet-derived growth factor (PDGF) and transforming growth factor β (TGFβ), which can stimulate the hepatic stellate cells (HSCs) and fibroblasts. The activation of HSCs by cytokines and other mediators is considered as a central event in the pathophysiology of liver fibrosis. Activated HSCs/myofibroblasts are the major source of ECM molecules, which comprise collagens, non-collagenous glycoproteins, proteoglycans, and glycoaminoglycans [2].

Oxidative stress is long known to be involved in the pathogenesis of hepatic fibrosis. It was shown that certain lipid peroxidation products induce genetic overexpression of fibrogenic cytokines and increase the synthesis of collagen by initiating the activation of HSCs [2]. On the other hand, it was demonstrated that antioxidant supplementation was able to suppress procollagen I messenger RNA (mRNA) overexpression and collagen deposition in fibrotic livers [3].

Evidence for the effectiveness against fibrosis is available for several plant-derived antioxidants, mainly polyphenols and flavonoids [4,5,6,7]. One such substance is silymarin, which demonstrates potent antioxidant and anti-inflammatory activity and which was proven to be effective in the treatment of chronic liver diseases [8].

Chokeberry (*Aronia melanocarpa* (Michx.) Elliott) fruit is a rich source of natural antioxidants. The content of polyphenolic compounds such as anthocyanins and proanthocyanidins in chokeberry is significantly higher than in other berries [9]. Moreover, aronia berries demonstrate the highest antioxidant capacity among other fruit investigated so far, as measured in some in vitro assays [10]. Numerous pharmacological activities of chokeberry were demonstrated by in vitro and in vivo studies, as reviewed by Kokotkiewicz et al. [11] and recently updated in the survey by Jurikova et al. [12].

Chokeberry extracts exert high antioxidant potential, which is comparable to that of prednisolone [11]. Consistently, their anti-inflammatory properties were documented in numerous experimental models, e.g., inhibition of NO production in LPS-induced RAW macrophages [13], inhibition of TNFα, IL-6, and IL-8 in human peripheral monocytes, inhibition of NF-κB activation in RAW macrophages [14], and inhibition of IL-6 in mouse splenocytes [15].

Antioxidant and anti-inflammatory properties of chokeberry contribute to the prevention of obesity and metabolic syndrome, as well as an antidiabetic effect. In rats fed a fructose-rich diet, chokeberry extract caused a decrease in epididymal fat, blood glucose, triglycerides, and LDL cholesterol, as well as plasma TNFα and IL-6 [16]. Hypoglycemic and hypolipidemic effects of chokeberry juice were also demonstrated in streptozotocin-induced diabetic rats [17]. Kim et al. [18] reported a decrease in the expression of genes involved in lipid metabolism and lipoprotein assembly in Caco-2 cells incubated with chokeberry extract [18]. Obese mice treated with *A. melanocarpa* extract showed a decrease in serum triglycerides and LDL cholesterol, as well as improved insulin sensitivity [19].

The above-mentioned findings were confirmed in patients suffering from metabolic syndrome. Chokeberry juice caused an improvement in their health status as assessed by the decrease in blood pressure, improved antioxidant status, reduction in triglyceride and LDL cholesterol levels, decrease in the activity of angiotensin1 converting enzyme, decrease in the serum activity of endotelin1, and decrease in body fat content [11,12].

The improvement in lipid metabolism and the anti-hypertensive effect of chokeberry are translated to its cardioprotective action. Additionally, chokeberry was shown to inhibit platelet aggregation [12] and to cause relaxation in porcine coronary arteries via the stimulation of endothelial NO formation [20]. Daskalova et al. [21] presented direct evidence of a cardioprotective effect in rats in which chokeberry diminished atherogenic changes in the aorta and coronary arteries [21]. The efficacy of chokeberry in cardioprotection was demonstrated in patients who survived myocardial infarction. Their treatment with chokeberry extract resulted in a marked decrease in blood pressure and in plasma level of angiotensin1 converting enzyme activity [22].

The chemopreventive and anticancer potential of chokeberry can be suggested on the basis of numerous in vitro experiments demonstrating the inhibition of the growth of various cell lines by chokeberry extracts, e.g., human leukemia, breast, colon, and cervical cancer cells, as well as murine leukemia and embryonic cancer stem cells [11,12]. The only report referring to the anticancer action of chokeberry in vivo was published by Lala et al. who demonstrated an inhibition of cellular proliferation induced by azoxymethane in rats [23].

The gastroprotective activity of chokeberry was demonstrated in rats with indomethacin- or ethanol-induced gastric lesions [12]. The hepatotoxicity of carbon tetrachloride, aminopyrine, and cadmium chloride in rodents was distinctly counteracted by the administration of chokeberry extract [11]. The bacteriostatic action of chokeberry juice was demonstrated in Gram-positive and Gram-negative bacteria, and its efficiency against various subtypes of influenza viruses was evidenced [12]. Additionally, chokeberry extract was shown to suppress endotoxin-induced uveitis in rats [24], and to improve the function of seminal vesicles in infertile men [25].

Recently, a new area of chokeberry pharmacological activity was revealed, namely, cognitive-enhancing [26] and beneficial behavioral effects [27] in rats drinking chokeberry juice, as well as attenuation of aging-related degenerative changes in the brain of mice treated with anthocyanins from chokeberry [28].

The hepatoprotective activity of chokeberry reported by Valcheva-Kuzmanova et al. in rats challenged with a single dose of CCl_4_ was confirmed in our studies [29]. We found that pretreatment with natural chokeberry juice attenuated acute liver injury induced in rats by a single dose of CCl_4_ or *N*-nitrosodiethylamine [30].

However, no data concerning the role of chokeberry in chronic liver impairment are reported so far in the available literature. The current study was undertaken to evaluate the potential antifibrotic effect of chokeberry juice and to compare its efficiency with that of known hepatoprotective agent silymarin.

## 2. Results

### 2.1. Antioxidant Status Parameters

The effect of chokeberry juice on microsomal lipid peroxidation in the liver of rats treated chronically with CCl_4_ is shown in Table 1. The carbon tetrachloride-induced increase in lipid peroxidation, by 48%, was attenuated by 55% following a simultaneous treatment with juice. A similar effect, i.e., a 48% decrease in lipid peroxidation, was observed in the group receiving silymarin + CCl_4_. The levels of TBARS in the liver of rats treated with hepatotoxin and either substance tested were lower than those in controls. Moreover, silymarin alone reduced the basal concentration of TBARS in rats not treated with CCl_4_ by 33%.

Treatment with CCl_4_ decreased the activities of hepatic antioxidant enzymes by 16%–78%; this alteration was not significant for glutathione reductase (Table 1).

In the A + T group (see Table 1), an increase in the activity of SOD by 71% and GR by 19% was observed. The effect of silymarin was a little better since the activities of SOD, GPx, and GR in the S + T group were enhanced by 174%, 25%, and 19%, respectively. The activity of GST was further decreased in the S + T group as compared to that in rats treated with CCl_4_ alone. A similar reduction in GST activity was caused by chokeberry juice alone as compared to controls. Hepatic reduced glutathione was not changed in any of the experimental groups (data not shown).

### 2.2. Clinical Chemistry, Hydroxyproline Concentration, and Gene Expression

The activities of serum hepatic enzymes were distinctly elevated, 3–51-fold, in rats treated with CCl_4_ (Table 2).

Protection against CCl_4_-induced hepatotoxicity by chokeberry juice was suggested by a decrease in the activities of AST, LDH, GGT, and SDH by 43%, 55%, 18%, and 44%, respectively. Silymarin caused a decrease in AST, LDH, and GGT activities by 55%, 22%, and 45%, respectively. Neither chokeberry juice nor silymarin reversed the CCl_4_-induced elevation in ALT activity. Treatment with silymarin alone resulted in a 54% reduction in SDH activity as compared to control (Table 2). In rats treated with CCl_4_, chokeberry juice or silymarin similarly affected elevated serum concentrations of bilirubin and cholesterol causing a decrease in both parameters by approximately 30% and 10%, respectively (Table 2).

**Figure 1:**
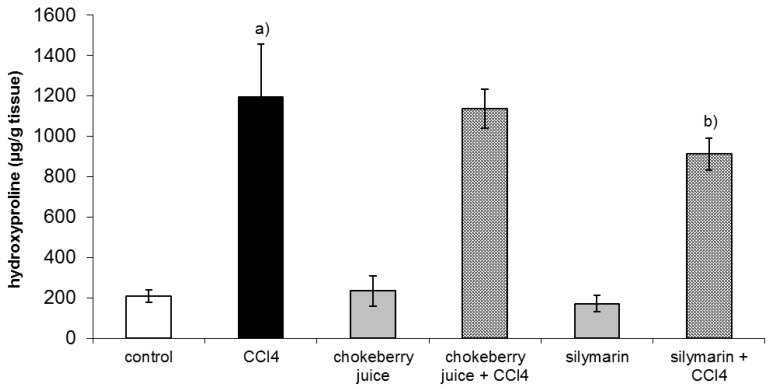
Effect of chokeberry juice or silymarin on hydroxyproline content in the liver of rats treated with CCl_4_. Results are means ± SD, *n* = 8. ^a)^ significantly different from control, *p* < 0.05; ^b)^ significantly different from CCl_4_-treated rats, *p* < 0.05.

After six week of treatment with CCl_4_ hepatic hydroxyproline, a marker of collagen content was about fivefold higher than that in controls. Co-treatment with chokeberry failed to diminish hydroxyproline level, while silymarin administration resulted in a 24% decrease in hydroxyproline content as compared to the CCl_4_-treated group (Figure 1).

**Figure 2:**
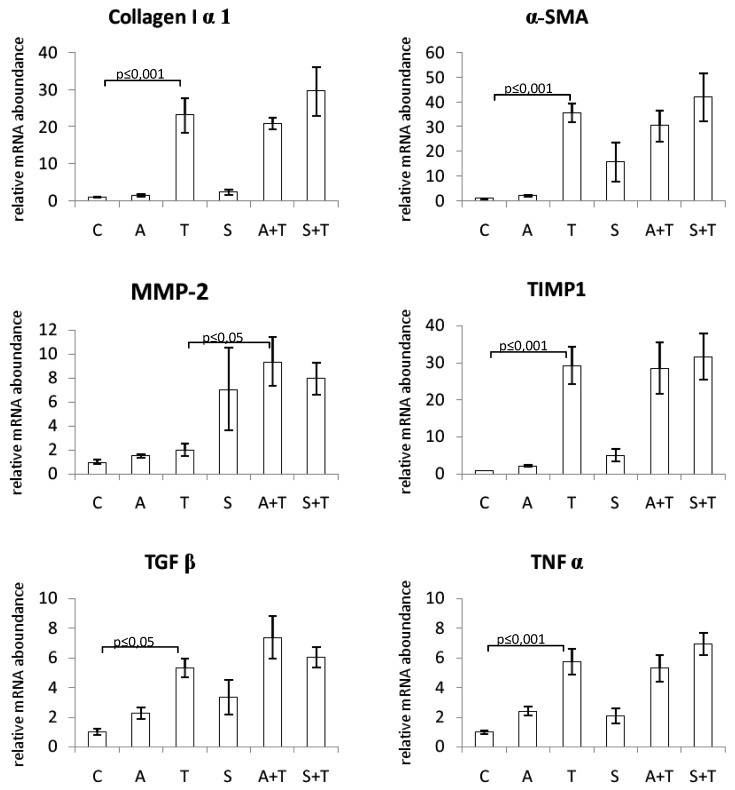
Effect of chokeberry juice or silymarin on the expression of fibrosis-related genes in the liver of rats treated with CCl_4_. Treatment groups: (C) controls; (A) chokeberry juice; (T) CCl_4_; (S) silymarin; (A + T) chokeberry juice + CCl_4_; (S + T) silymarin + CCl_4_.

The mRNA expression levels of procollagen I, α-SMA, TIMP-1, TGFβ, and TNFα were significantly increased in the liver of rats treated with CCl_4_. Neither chokeberry juice nor silymarin modulated the levels of these transcripts. MMP-2 mRNA expression was not affected by CCl_4_ alone; however, combined treatment with CCl_4_ and chokeberry resulted in its increased expression as compared to CCl_4_-administered rats (Figure 2).

### 2.3. Morphology and Morphometry

**Figure 3:**
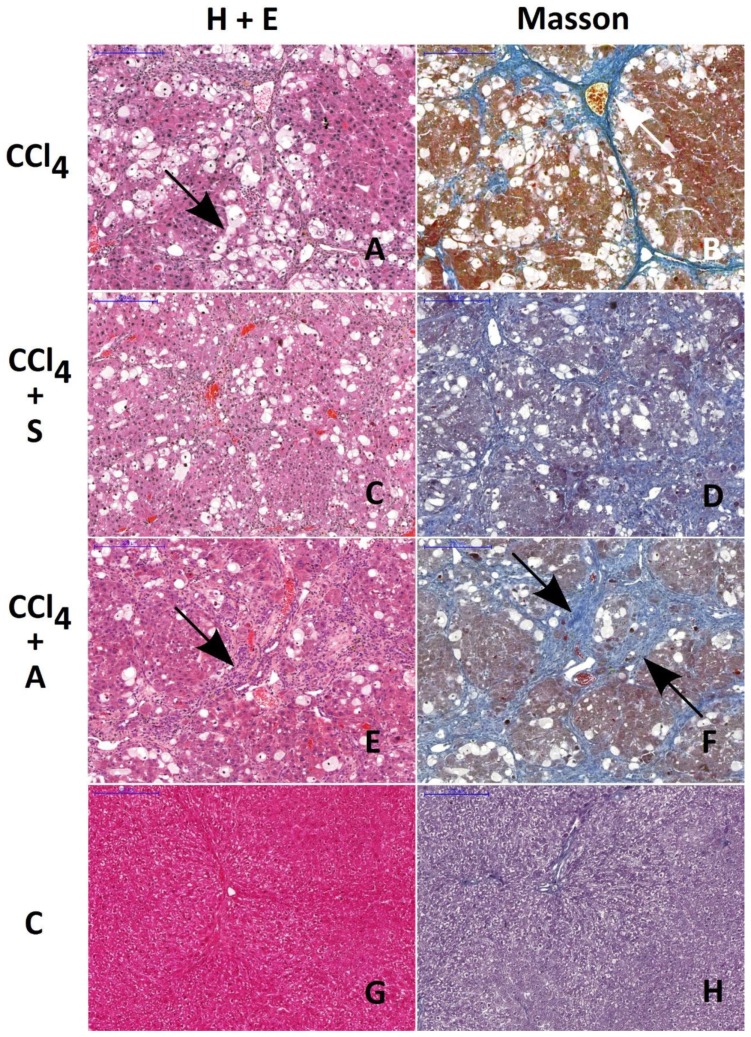
Histological evaluation of morphological changes in livers of rats treated with CCl_4_ (**A** and **B**), CCl_4_ + silymarin (**C** and **D**), and CCl_4_ + chokeberry (**E** and **F**). Control livers are presented in figures (**G** and **H)**. H&E and Masson staining. Note the similar extent of fibrosis in rats treated with CCl_4_ and with CCl_4_ + chokeberry (arrows on **A**, **B**, **E**, and **F**). The number of vacuolated hepatocytes is significantly reduced in CCl_4_ + chokeberry-treated rats (**E** and **F**) as compared to CCl_4_-treated animals. This alteration is accompanied by the increased expression of lymphocyte infiltration. Scale bar: 200 µm.

All morphological findings are presented in Figure 3 and Table 3.

The ratio of the area affected by fibrosis to the total area of the liver was very similar in CCl_4_- and CCl_4_ + chokeberry-treated rats, amounting to 0.18 ± 0.03 (Figure 3B) and 0.23 ± 0.05 (Figure 3F), respectively. In rats treated with CCl_4_ + silymarin, the ratio 0.09 ± 0.01 was significantly lower (*p* = 0.036) as compared to the CCl_4_-treated group (Figure 3D); however, it was still higher than that in controls, 0.04 ± 0.01 (Figure 3H).

The extent of hepatocyte damage, defined by the number of hepatocytes with vacuolization, amounted to 208 ± 23 vacuolated hepatocytes (vh) per mm^2^ in the CCl_4_ group (Figure 3A), 65 ± 14 vh/mm_2_ in the A + T group (Figure 3E), and 179 ± 39 vh/mm^2^ in the S + T group. The decrease in the number of vacuolated cells per mm^2^ was statistically significant only in the A + T group as compared to the CCl_4_-treated rats (*p* = 0.029). In the control group (Figure 3G), as well as in groups treated with the substances tested alone (data not shown), no cells with vacuolization were detected.

## 3. Discussion

We compared the potential effects of two substances, chokeberry juice and silymarin, on chronic liver injury in rats. Silymarin served as a reference substance since it is considered to be a hepatoprotective and antifibrotic agent [8]. To test the contribution of antioxidant activity of the substances tested to the preventing effect on liver fibrosis, some parameters of oxidative stress were assayed.

CCl_4_-induced oxidative stress was reflected by the increase in the level of hepatic microsomal lipid peroxidation (LPO). Both substances diminished LPO to a similar degree. The antioxidant enzyme activities were significantly suppressed by the CCl_4_ challenge. Only some of these enzymes were partly restored by co-treatment with chokeberry juice or silymarin. The protective effect of silymarin on antioxidant enzymes in chronic liver injury was not extensively reported in the literature except for our previous work in which silymarin was shown to attenuate the decrease in SOD, CAT GPx, and GR activities in the liver of fibrotic rats [31]. In the current study, both chokeberry juice and silymarin alone caused a slight decrease in the GST activity, while the effect of the latter was not statistically significant. This could be attributed to the fact that some flavonoids and silybin, a major constituent of silymarin, were found to be inhibitors of GST in vitro [32,33]. The further decrease in GST activity in rats treated simultaneously with prooxidant and silymarin might be due to the combination of the inhibitory effect of silymarin and the damage of the enzyme by free radicals generated by CCl_4_.

Hepatic glutathione does not seem to play a significant role in the process of fibrosis in this model. The depleted concentration of GSH in fibrotic rats was consistent with our previous findings and other authors’ observations [31,32,33,34,35,36]. Neither substance tested in our experiment affected this parameter.

Hydroxyproline is a generally accepted marker of collagen accumulation in the liver. In our study, silymarin decreased the content of hydroxyproline by 30% which was consistent with the report of Mourelle et al., who found the same reduction in hepatic collagen in rats co-treated with CCl_4_ and silymarin (50 mg/kg/day) for eight weeks [37]. In our previous work, in the same model of rat liver fibrosis, silymarin caused a 60% decrease in hepatic hydroxyproline content [31]. A decrease in collagen accumulation was observed in rats with another model of fibrosis induced by complete bile duct occlusion (BDO) and administered silymarin at a dose 50 mg/kg/day for six weeks [38,39]. However, the effects of silymarin on liver fibrosis are not consistent since, in the same model of liver injury, Muriel and Moreno did not demonstrate the prevention of lipid peroxidation and collagen accumulation by silymarin administration [40].

Proanthocyanidins, the main components of chokeberry juice, were reported to demonstrate free radical scavenging and antioxidant properties [41]. The antifibrotic activity of grape-derived proanthocyanidins was evidenced by the reduction of liver collagen content and improvement in liver histology in rats with BDO- or *N*-nitrosodimethylamine-induced fibrosis [42,43,44]. Despite the high content of proanthocyanidins, chokeberry juice did not decrease the collagen content in the livers of CCl_4_-treated rats.

Histological examination revealed that only silymarin was able to reduce the extent of connective tissue fibrosis in the liver of rats treated with CCl_4_, which is consistent with the above-mentioned findings concerning the content of hepatic collagen. On the other hand, chokeberry significantly reduced the number of vacuolated hepatocytes, an effect which was not observed in rats treated with silymarin prior to CCl_4_ challenge. It can be suggested that chokeberry-mediated protection of hepatocytes might be due to the activation of the immunologic system as evidenced by the enhanced infiltration of lymphocytes in the injured areas of the liver.

Routine liver function markers, i.e., serum hepatic enzymes and bilirubin, are not directly related to fibrogenesis but they reflect the comparable protective effect of both substances tested on chronic liver injury evoked by CCl_4_.

To assess the possible molecular mechanism of the antifibrotic effect of substances tested, we examined the changes in expression of several fibrosis-associated genes in the liver tissue. α-SMA is known to be a specific marker of HSC activation, and its mRNA expression was markedly increased as a result of the treatment of rats with CCl_4_. It is also known that liver injury elevates some cytokines which can activate hepatic stellate cells to produce collagen and cause liver fibrosis. TGFβ was shown to be an essential pro-fibrogenic mediator, which plays a key role in the activation of stellate cells and, during liver repair, limits the proliferative response of hepatocytes and increases the production of extracellular matrix proteins, especially type I collagen [45]. Hence, in our study, the levels of TGFβ and collagen I mRNA were markedly elevated in rats treated with CCl_4_. Matrix metalloproteinases (MMPs) comprise a family of zinc-dependent enzymes that specifically degrade ECM components. During fibrogenesis, the expression of some MMPs increases. The activity of MMPs is regulated by tissue inhibitors of metalloproteinases (TIMPs). Injured liver tissue expresses more TIMP-1, which results in the enhanced accumulation of fibrils [45]. In the current study, we demonstrated that the expression of TIMP-1 mRNA in the liver was significantly enhanced following the treatment with CCl_4_, in contrast to MMP-2 expression which was not affected. Oxidative stress evoked by CCl_4_ causes necrosis of hepatocytes and induces inflammation. Since TNFα is a major mediator of inflammation, we also examined its expression and observed a marked increase in TNFα mRNA in rats challenged with CCl_4_. However, we found that neither substance tested affected mRNA levels of the above-mentioned molecules responsible for the production of extracellular matrix components and their decomposition. The only exception was MMP-2, whose expression was increased by silymarin alone, as well as by combined treatment with either substance and carbon tetrachloride. This effect is considered beneficial since matrix metalloproteinases are responsible for the degradation of ECM components. Consistently, the enhanced MMP-2 expression was paralleled by a decrease in fibrotic area and collagen content in the same groups of rats.

In the available literature, we found a single report describing changes in gene expression induced by silymarin in fibrotic rats [39]. The authors demonstrated significant downregulation of mRNA levels of procollagen I, TIMP 1, and TGFβ1 in rats with BDO-induced liver fibrosis administered silymarin.

It could be suggested that the effect of silymarin on fibrosis-related gene expression depends on the model of fibrogenesis. Some authors revealed that oxidative stress does not play a key role in liver damage produced by bile duct occlusion. BDO-induced liver injury is essentially devoid of necrosis and inflammation [38]. However, the hepatotoxicity of CCl_4_ involves oxidative stress accompanied by a strong necroinflammatory component, and it seems that neither silymarin nor chokeberry juice is efficient enough in modulating some gene expression in this model of chronic liver injury. There are examples of substances such as colchicine, malotilate, and d-penicillamine which are effective in models of radical-induced liver fibrosis, but which do not demonstrate antifibrotic activity in fibrosis where free radicals play a minor role [38]. Another example demonstrating that antifibrotic efficiency depends on a model of fibrogenesis is halofuginone. This antiparasitic drug was found to improve recovery from thioacetamide-induced liver fibrosis, although, in other studies, a harmful effect of this compound in a model of biliary obstruction was observed [46].

## 4. Materials and Methods

### 4.1. Chemicals

Chokeberry juice was purchased from PTHU ECOAR (Lewin Kłodzki, Poland). The content of active compounds (mg/100 mL) was determined by HPLC according to the method described by Oszmiański and Wojdyło [9], as presented in Table 4. The total content of polyphenolic compounds amounted to 315.9 mg/100 mL.

The chemicals used were purchased from Sigma Aldrich, Poland. Kits for serum parameters assays, alanine aminotransferase (ALT), aspartate aminotransferase (AST), alkaline phosphatase (AP), sorbitol dehydrogenase (SDH) activities, total cholesterol, total bilirubin, and albumin concentration, were from Pointe Scientific Poland, and that for γ-glutamyltransferase (GGT) activity was from Alpha Diagnostics Poland. Silymarin (standardized for silybin; 30%) was purchased from Sigma Aldrich (Poznań, Poland).

For reverse transcription PCR (RT-PCR), the Transcriptor First Strand complementary DNA (cDNA) Synthesis kit was used (Roche, Mannheim, Germany). The LightCycler^®^ 480 Probes Master kit for real-time quantitative PCR (RQ-PCR) analysis was provided by Roche (Mannheim, Germany).

### 4.2. Experimental Design

Male Wistar rats weighing 200 ± 10 g were bred in the Department of Toxicology, Poznan University of Medical Sciences. Animals were randomly divided into six experimental groups, eight animals each. The rats were housed in plastic cages (Techniplast) and maintained in a temperature- (21 ± 2 °C), humidity- (40%–50%), ventilation-, and light-controlled (12-h light/dark cycle) environment. They were given free access to commercial rat chow and tap water. In the three groups labeled T, A + T, and S + T, liver damage was induced by CCl_4_ given per os at a dose of 0.7 mL/kg body weight (b.w.), two days a week, for six weeks. Group A + T was treated simultaneously with chokeberry juice 10 mL/kg b.w./day for six weeks. According to the same protocol group, T + S was given silymarin (100 mg/kg b.w./day) used as a reference substance. Groups A and S received only juice or silymarin, respectively, for six weeks in the same amounts. Group C served as a control and was given water. Both substances were administered intragastrically. Blood was collected by intracardiac puncture under ketamine terminal anesthesia, and serum was separated. Livers were removed, perfused with ice-cold 1.15% KCl, and homogenized in buffered sucrose solution (Tris, pH 7.55). Microsomal and cytosol fractions were prepared via differential centrifugation according to a standard procedure. Protein concentration in the fractions was determined using Folin–Ciocalteu reagent.

The experimental protocol was approved by the Local Animal Ethics Committee in Poznań.

### 4.3. Biochemical Assays

Microsomal lipid peroxidation (LPO) in the liver was evaluated by measuring thiobarbituric acid reactive substances (TBARS) [47]. Reduced glutathione (GSH) level was assayed in the liver homogenate prepared in phosphate buffer (pH 7.4) by the Ellman’s method [48]. Antioxidant enzymes were assayed in the cytosol fraction. Glutathione peroxidase (GPx) activity was determined according to Mohandas et al. [49]. Hydrogen peroxide was used as a substrate. Glutathione reductase (GR) was assayed by measuring NADPH oxidation at 340 nm using oxidized glutathione as a substrate [49]. Glutathione *S*-transferase (GST) activity measurement was based on the spectrophotometric determination of a 1-chloro-2,4-dinitrobenzene (CDNB) conjugate formed in a GSH-coupled reaction [49]. Catalase (CAT) activity was determined by measuring the rate of H_2_O_2_ reduction [50]. The determination of superoxide dismutase (SOD) activity was based on the inhibition of spontaneous epinephrine oxidation [50]. Serum parameters were assayed using commercially available kits as specified in the Chemicals section. Liver collagen concentration was determined by measuring hydroxyproline content in fresh liver samples after digestion with 6 M hydrochloric acid for 20 h at 100 °C [31].

### 4.4. Real-Time Quantitative PCR (RQ-PCR) Analysis

Total RNA was isolated according to the method of Chomczynski and Sacchi [51]. The RNA concentration was quantified by measuring the optical density (OD) at 260 nm, and integrity was confirmed by denaturing agarose gel electrophoresis. RNA samples were treated with DNase I and reverse-transcribed into cDNA using oligo-dT primers.

Dual-color RQ-PCR was carried out by LightCycler^®^ Instrument 480 Multiwell Plate 96 (Roche, Mannheim, Germany) using the LightCycler^®^ 480 Probes Master kit. Target cDNA was quantified using the relative quantification method. The quantity of procollagen I, α-SMA, TIMP-1, MMP-2, TGFβ, and TNFα (Universal ProbeLibrary Set, Rat, Roche, Mannheim, Germany) in each sample was standardized by GAPD and ACTB transcript levels (Universal ProbeLibrary Rat GAPD, ACTB Gene Assay, Roche, Mannheim, Germany). For amplification, 1 µL of the total (20 µL) cDNA solution was added to 9 µL of the LightCycler^®^ 480 Probes Master kit from Roche (Mannheim, Germany), as well as primers and probes for procollagen I, α-SMA, TIMP-1, MMP-2, TGFβ, and TNFα (see Table S1, Supplementary Materials). In the case of the negative control, cDNA was not added.

### 4.5. Histological Examination

All liver samples were fixed in Bouin’s solution, embedded in paraffin, and cut into 5–6-µm-thin subsequent sections. The slides were stained with hematoxylin and eosin and Masson trichrome for visualization of morphological changes and the detection of collagen fibers. The ratio of the area affected by fibrosis and infiltrated by lymphocytes to the total area of the liver section, as well as the density of vacuolated hepatocytes, was assessed.

All tissue images were taken using a charge-coupled device (CCD) camera connected to a Nikon Digital Sight S-U1microscope. Variations within liver tissue architecture were evaluated and calculated using Micro Image v.4.0 software (Olympus, MS Windows XP); then, the images were labeled and assembled as plates using Adobe Photoshop 7.0. Ten sections of each tissue were examined by two independent researchers. In 100 and 200 high-power fields of each section, the number of vacuolated hepatocytes (per square millimeter), the fibrotic area, and the surface occupied by lymphocyte infiltration (defined as the ratio of fibrosis or infiltration area to the total examined liver surface) were assessed.

### 4.6. Statistical Analysis

The GraphPad InStat statistical package (version 3) was used. The data were expressed as means ± SD. One-way analysis of variance (ANOVA) followed by the Student–Newman–Keuls test for multiple comparisons was used for biochemical parameters and mRNA concentration. Differences in the observed density of vacuolated hepatocytes and the percentage of fibrotic or lymphocyte infiltrated areas between the treated and control rats were analyzed by ANOVA test; *p* < 0.05 was considered to be the limit of significance.

## 5. Conclusions

Summing up, chokeberry juice did not demonstrate an antifibrotic effect in the applied experimental model of fibrosis, and the effect of the known antifibrotic agent, silymarin, was not distinct. Neither substance tested affected the expression of selected fibrosis-related genes. Thus, the antioxidant activity shown by chokeberry juice was not translated into an attenuation of liver fibrosis. However, histological examination revealed the beneficial effect of chokeberry as evidenced by the reduced number of vacuolated hepatocytes and enhanced infiltration of lymphocytes in the injured areas of the liver.

## Figures and Tables

**Table 1:** Effect of chokeberry juice or silymarin on hepatic lipid peroxidation and antioxidant enzymes in the liver of rats treated with carbon tetrachloride.

Treatment	LPO	Enzymes				
		SOD	CAT	GPx	GR	GST
Controls	0.21 ± 0.03	32.9 ± 4.8	4.13 ± 0.33	821.0 ± 83.0	54.4 ± 6.3	903.3 ± 63.9
CCl_4_	0.31 ± 0.06 ^a^	7.2 ± 1.2 ^a^	2.12 ±0.34 ^a^	427.7 ± 48.9 ^a^	46.0 ± 5.3	585.8 ± 62.4 ^a^
(T)	(↑48%)	(↓78%)	(↓49%)	(↓48%)	(↓16%)	(↓35%)
Chokeberry	0. 21 ± 0.02	29.1 ± 3.1	4.83 ± 0.43 ^a^	882.2 ± 65.3 ^a^	44.8 ± 7.0	755.6 ± 29.4 ^a^
juice	—	(↓12%)	(↑17%)	—	(↓18%)	(↓16%)
(A)						
–						
CCl_4_ +	0.14 ± 0.03 ^b^	12.3 ± 2.4 ^b^	1.79 ± 0.27	440.5 ± 41.4	54.9 ± 3.7 ^b^	576.7 ± 52.2
chokeberry	(↓55%)	(↑71%)	(↓16%)	(↑19%)	(↑19%)	—
juice						
(A + T)						
Silymarin	0.14 ± 0,02 ^a^	35.0 ± 6.1	4.73 ± 0.25^a^	978.2 ± 84.1 ^a^	51.1 ± 7.4	820.2 ± 57.3
(S)	(↓33%)	—	(↑15%)	(↑19%)	—	—
CCl_4_ +	0.16 ± 0.03 ^b^	19.7 ± 4.7 ^b^	2.27 ± 0.21	535.6 ± 46.0 ^b^	54.7 ± 3.1 ^b^	472.4 ± 60.3 ^b^
silymarin	(↓48%)	(↑174%)	—	(↑25%)	(↑19%)	(↓19%)
(S+T)						

Results are means ± SD, *n* = 8. Control rats were administered water. ^a^ The juice-, silymarin-, and CCl_4_-treated groups are compared with controls, *p* < 0.05. ^b^ The CCl_4_-treated group is compared with the juice + CCl_4_- and silymarin + CCl_4_-treated groups, *p* < 0.05. Values in parentheses express the percentage of statistically significant change. The level of lipid peroxidation is expressed as nmol TBARS/min/mg protein. The activity of antioxidant enzymes is expressed as follows: SOD, CAT—U/mg protein; GPx, GR—nmol NADPH/min/mg protein; GST—nmol CDNB/min/mg protein.

**Table 2:** Effect of chokeberry juice or silymarin on serum parameters in rats treated with carbon tetrachloride.

			Treatment			
Parameters	Controls	CCl_4_	Chokeberry	CCl_4_ +	Silymarin	CCl_4_ +
			Juice	Chokeberry		Silymarin
		(T)	(A)	Juice	(S)	(S + T)
				(A + T)		
ALT	60.1 ± 10.2	886 ± 95.7 ^a^	56.3 ± 9.4	838 ± 104	49.4 ± 4.5	881 ± 89.0
(IU/L)		(↑1374%)				
AST	78.7 ± 12.3	3401 ± 319^a^	86.6 ± 4.0	1722 ± 194 ^b^	76.2 ± 15.7	1378 ± 216 ^b^
(IU/L)		(↑3766%)		(↓49%)		(↓55%)
ALP	190.9 ± 22.7	844 ± 84.1 ^a^	209.0 ± 35.7	772 ± 54.6	222 ± 16.2	820 ± 72.2
(IU/L)		(↑342%)				
LDH	183.0 ± 20.0	804 ± 96.3 ^a^	161.7 ± 24.4	364 ± 31.5 ^b^	154 ± 28.0	624 ± 55.9 ^b^
(IU/L)		(↑339%)		(↓55%)		(↓22%)
GGT	10.1 ± 1.0	36.9 ± 6.5 ^a^	8.2 ± 1.2	30.2 ± 2.0 ^b^	8.7 ± 1.0	20.3 ± 2.4 ^b^
(IU/L)		(↑364%)		(↓18%)		(↓45%)
SDH	7.9 ± 1.2	415 ± 67.5 ^a^	6.9 ± 1.4	234 ± 36.2 ^b^	3.9 ± 0.7	358 ± 38.3
(IU/L)		(↑5136%)		(↓44%)		
Bilirubin	0.32 ± 0.1	2.17 ± 0,2 ^a^	0.38 ± 0.1	1.48 ± 0.19 ^b^	0.29 ± 0.1	1.51 ± 0.2 ^b^
(mg/dL)		(↑584%)		(↓32%)		(↓30%)
Cholesterol	92.7 ± 4.3	123 ± 7.3 ^a^	83.6 ± 5.0 ^a^	112 ± 4.3 ^b^	83.8 ± 4.5 ^a^	109 ± 7.3 ^b^
(mg/dL)		(↑33%)	(↓10%)	(↓9%)	(↓10%)	(↓11%)

Results are means ± SD, *n* = 8. Control rats were administered water. ^a^ The juice-, silymarin-, and CCl_4_-treated groups are compared with controls, *p* < 0.05. ^b^ The CCl_4_-treated group is compared with the juice + CCl_4_- and silymarin + CCl_4_-treated groups, *p* < 0.05. Values in parentheses indicate the percentage of statistically significant change.

**Table 3:** Effect of chokeberry juice or silymarin on the level of liver fibrosis, lymphocyte infiltration, and density of vacuolated hepatocytes.

Parameters			Treatment			
	Controls	CCl_4_	Chokeberry	CCl_4_ +	Silymarin	CCl_4_ +
			Juice	Chokeberry		Silymarin
		(T)	(A)	Juice	(S)	(S + T)
				(A + T)		
Fibrosis	0.04 ± 0.01	0.18 ± 0.03	0.04 ± 0.01	0.23 ± 0.05	0.04 ± 0.01	0.09 ± 0.01 ^a,b^
Infiltration	-	4.6 ± 2.2	-	14.3 ± 6.6 ^c^	-	6.2 ± 2.2
Vacuolization	-	208 ± 23	-	65 ± 14 ^d^	-	179 ± 39

Results are means ± SD, *n* = 8. Control rats were administered water. Fibrosis = the ratio of the area affected by fibrosis to the total area examined. Infiltration = the ratio of area infiltrated by lymphocytes to the total area examined. Vacuolization = number of vacuolated hepatocytes (density) per 1 mm^2^. “-” absence of the trait; ^a^ S + T group vs. CCl_4_-treated animals, *p* < 0.036; ^b^ S + T group vs. controls, *p* < 0.001; ^c,d^ A + T group vs. CCl_4_-treated rats, *p* = 0.029.

**Table 4:** Content of phenolic compounds in chokeberry juice (mg/100 mL).

Compounds	
Polymeric procyanidins	224.90
Neochlorogenic acid	46.40
Chlorogenic acid	35.40
Quercetin 3-rutinoside	1.85
Cyanidin 3-galactoside	1.62
Quercetin 3-galactoside	1.42
Quercetin 3-glucoside	1.10
Quercetin 3-vicianoside	1.10
Quercetin 3-robinobioside	1.05
Cyanidin 3-arabinoside	0.66
Cyanidin 3-glucoside	0.16
Cyanidin 3-xyloside	0.20
Total	315.90

## Supplementary Materials

Table S1. Oligonucleotide sequences and probe number used for RQ-PCR analysis.
