# Supplementary material for: Assessment of Hepatoprotective Effect of Chokeberry Juice in Rats Treated Chronically with Carbon Tetrachloride
Source: Molecules. 2020 Mar 11;25(6):1268. doi: 10.3390/molecules25061268 (PMC7144002; doi:10.3390/molecules25061268)
Supplement: Supplementary file 1 [file molecules-25-01268-s001.pdf]

Table S1. Oligonucleotide sequences and probe number used for RQ-PCR analysis

Transcript	Sequence (5'-3' direction)	Probe number	NCBI number	Product size (bp)
TNFα	5'-CTCGAGTGACAAGCCCGTA-3'<br>5'-CCACTCCAGCTGCTCCTCT-3'	119	NM_012675.2	64 bp
TGFβ	5'-GAAGGACCTGGGTTGGAAGT-3'<br>5'-CAGACAGAAGTTGGCATGGTAG -3'	117	NM_021578.2	61 bp
MMP-2	5'-AGCGACCTCAGGGTGACA-3'<br>5'-CACTCCCCAGACCAATCGT-3'	58	NM_031054.2	67 bp
TIMP-1	5'-TTTCCGGTTCGCCTACAC-3'<br>5'-CGGTTCTGGGACTTGTGG-3'	110	NM_053819.1	66 bp
procollagen I	5'-TGCTTGAAGACCTATGTGGGTA-3'<br>5'-AAAGGCAGCATTTGGGGTAT-3'	113	NM_053304.1	71 bp
α-SMA	5'-TGCCATGTATGTGGCTATTCA-3'<br>5'-ACCAGTTGTACGTCCAGAAGC-3'	56	NM_031004.2	61 bp
